# Thermal tolerances of *Popenaias popeii* (Texas hornshell) and its host fish from the Rio Grande Basin, Texas

**DOI:** 10.1038/s41598-023-29460-9

**Published:** 2023-03-21

**Authors:** Xenia L. Rangaswami, Amanda M. Goldsmith, Jennifer M. Khan, Clinton R. Robertson, Roel R. Lopez, Charles R. Randklev

**Affiliations:** 1 Texas A&M Natural Resources Institute, 578 John Kimbrough Blvd. 2260 TAMU, College Station, TX 77843 USA; 2 Texas A&M AgriLife Research and Extension Center at Dallas, 17360 Coit Rd., Dallas, TX 75252 USA; 3 U.S. Fish and Wildlife Service, 2005 Northeast Green Oaks Boulevard, Suite 140, Arlington, TX 76006 USA; 4 Texas Parks and Wildlife Department, River Studies Program, 505 Staples Rd., Bldg. 1, San Marcos, TX 78666 USA

**Keywords:** Climate-change ecology, Conservation biology, Ecophysiology, Freshwater ecology

## Abstract

Freshwater mussels are particularly sensitive to hydrologic changes, including streamflow and temperature, resulting in global decline. The Devils River in south-central Texas harbors the endangered freshwater mussel *Popenaias popeii* (Unionidae; Texas hornshell). There is concern that water withdrawals from the underlying aquifer may be negatively impacting this species. To assess this risk, we evaluated upper thermal tolerances (LT05 and LT50) of larvae (glochidia) and juveniles from two sites. After being acclimated to 27 °C, glochidia were subjected to five experimental temperatures (30, 32, 34, 36, and 38 °C) and non-acclimated control (20 °C) for 12-h and 24-h while juveniles were subjected to three experimental temperatures (30, 32, and 36 °C) and non-acclimated control (20 °C) for 96-h. We overlaid tolerance estimates against in situ water temperature and discharge data to evaluate thermal exceedances. Additionally, we reviewed upper thermal tolerances of *P. popeii*’s presumed host fish (*Carpiodes carpio*, *Cyprinellas lutrensis*, and *Moxostoma congestum*) and their congeners. Stream temperatures only occasionally exceeded mussel LT05/50 and fish CLMax/LTMax, likely due to the Devils River’s large spring input, highlighting the importance of protecting spring flows. We provide a practical framework for assessing hydrological needs of aquatic ectotherms, including the parasite-host relationship, which can be used to optimize environmental management.

## Introduction

Stream temperature and flow shape the ecological integrity of riverine systems, and therefore changes to either factor can impact aquatic biota^1,2^. Specifically, changes to flow can lead to increases in stream temperatures, which, in turn, may exceed a species’ thermal optimum, causing sublethal and lethal impacts^3–5^. If these thermal impacts are frequent, species occupancy may be altered due to changes in population performance (i.e., growth, survivorship, and reproduction)^6^. Over time, changes in occupancy may lead to range shifts, and eventually extirpation followed by extinction^7–9^. Conservationists and natural resource managers have been acutely aware of the role stream temperature plays in the physiology of aquatic biota^10,11^, but have not fully appreciated the conservation implications of this relationship for a number of taxa. However, this is beginning to change given the threat of global climate change combined with increases in human water usage^12,13^.

Freshwater mussels (Unionidae) are considered one of the most imperiled faunal groups in the world, yet their decline is not fully understood, though it is thought to be partially rooted in stream temperature changes^14,15^. Mussels are particularly sensitive to stream temperature since they are sedentary ectotherms, and therefore reliant on ambient temperature to regulate physiological processes^16,17^. Although water temperature is positively correlated with growth^18^, changes in temperature beyond a thermal optimum can negatively affect metabolic and immune function and cause cellular damage^19,20^. These physiological responses should have consequences to mussel population performance^21^, which in turn shapes population persistence. Mussels also exhibit behavioral responses to water temperature. Zimmerman and Neves^22^ observed that elevated water temperatures of 25 °C resulted in decreased viability time for *Villosa iris* (rainbow mussel) and *Actinonalas pectorosa* (pheasantshell) glochidia compared to 0 and 10 °C. Unfortunately, little is known about mussel thermal tolerance, though this has started to change^23–25^.

In addition to being sedentary ectotherms, mussels are obligate ectoparasites, requiring a host for completion of their reproductive cycle and dispersal^26^. Similarly to mussels, fish have thermal optima, and exceedances of these thresholds can have lethal and sublethal impacts which affect population performance over time^21,27^. Changes to host fish occurrence and abundance could lead to periods when mussels are unable to reproduce because their hosts are absent. Consistent with the lack of thermal tolerance information for mussels, a significant knowledge gap exists regarding how elevated stream temperatures impact mussel-fish relationships.

Texas, located in the southwestern United States, is considered ground-zero for changes in streamflow and temperature due to predicted climate change and water usage^28–30^. Specifically, climate projections for south Texas indicate surface temperatures will increase 1.4–3.4 °C and precipitation will decrease almost tenfold by 2070 compared to average conditions from 1970 to 2000^31^. These changes are alarming given that streams within this region contain a number of rare and endemic species. The Devils River, a tributary of the Rio Grande, is one of the most pristine systems remaining in the state and harbors stronghold populations of multiple regionally endemic freshwater fishes and the federally endangered unionid *Popenaias popeii* (Texas hornshell). While declining streamflow and elevated temperatures are expected to increase in this system^29,31^, it is unknown how these changes will impact *P. popeii* since thermal tolerance data aren’t available to assess these risks.

The aim of this study was to evaluate the upper thermal tolerances of glochidial (larval) and juvenile life stages of *P. popeii* from two subpopulations (Grass Patch and Ruthies) within the Devils River in Texas. The specific objectives were to: (1) determine the upper thermal limits (LT05 and LT50) of glochidia and newly transformed juveniles across a range of experimental temperatures (30–38 °C) in standard acute (12-h, 24-h, and 96-h) laboratory tests; (2) overlay the resulting LT05 and LT50 thresholds onto in situ water temperature and discharge data from the Devils River to identify environmental flow bottlenecks; (3) review the thermal tolerances of *P. popeii*’s presumptive host fish; and (4) provide recommendations that can be used to inform water management practices focused on protecting instream flows for *P. popeii* and its hosts as well as other aquatic species.

## Methods

### Study area

The Devils River is located in the semiarid Edwards Plateau ecoregion of south-central Texas^32^. It is a spring-fed karst system, formed by porous limestone with sinkholes, which resides above the western Edwards-Trinity aquifer^33^. The Devils River receives less than 500 mm of annual precipitation, so baseflow is derived from the aquifer^33–35^. Surface runoff, especially during high precipitation events, and spring discharge into the riverbed are the main sources of recharge for the river^34^.

### Study species

*Popenaias popeii* is endemic to the Rio Grande Basin in Texas, New Mexico, and northern Mexico. It is currently listed as endangered in the U.S. under the Endangered Species Act of 1973^36^. There are five extant populations in the U.S., located in the Black River in New Mexico and the Pecos River, Devils River, Lower Canyons of the Rio Grande, and Lower Rio Grande near Laredo in Texas^37^. Its current range is estimated to be restricted to 21% of its presumptive historical range in the U.S., and its predicted occupancy within this range is 17% overall and 67% within the Devils River^38^. Declining flows and increasing water temperatures have been implicated as causal factors in *P. popeii*’s decline^37^.

### Collection and maintenance

**Figure 1:**
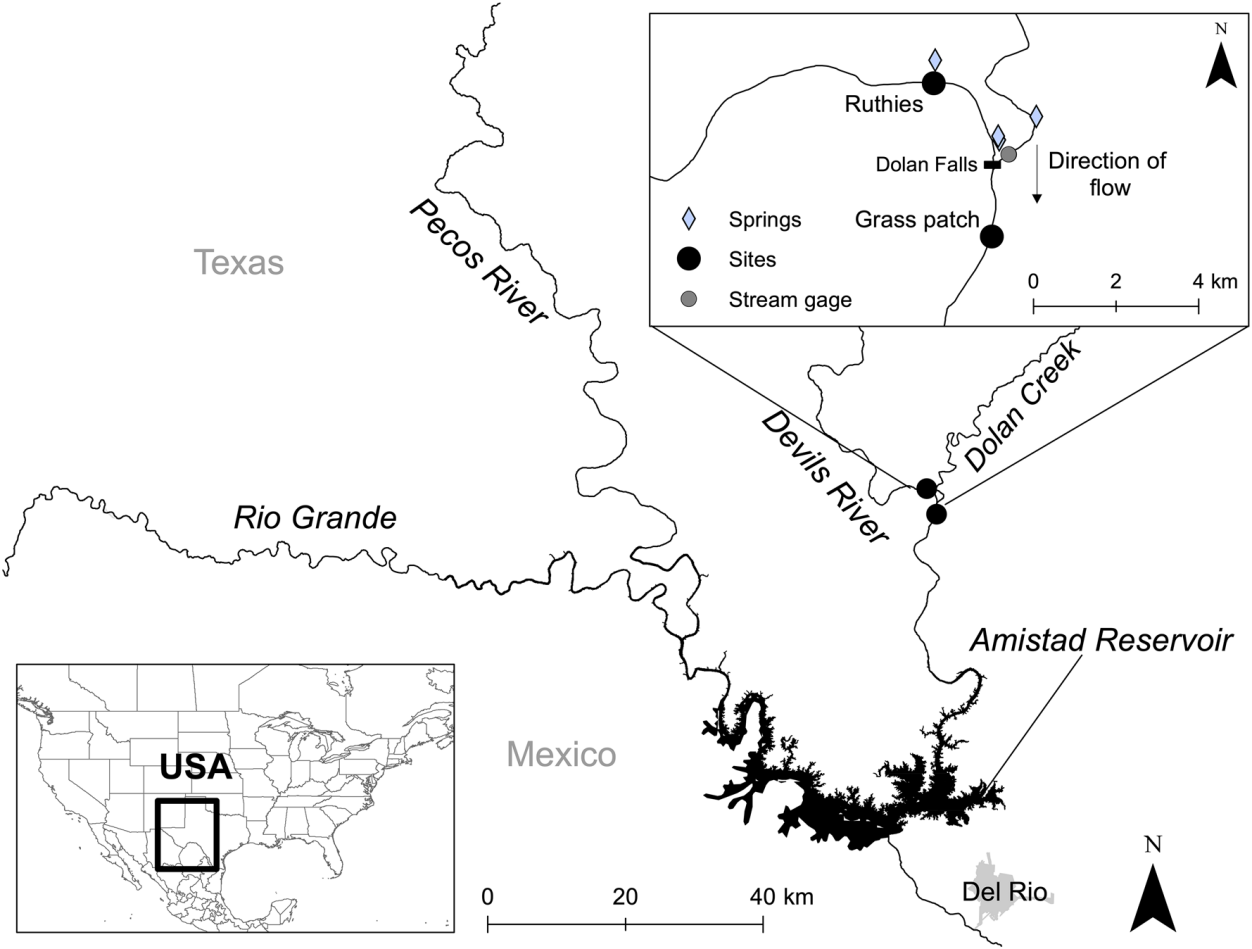
Map of study area. Blue diamonds denote springs, black circles denote sites, and the gray circle denotes the International Boundary and Water Commission (IBWC) gaging station. Gravid females of *Popenaias popeii* (Texas hornshell) were collected from two sites in the Devils River in Texas. Their glochidia were used in thermal experiments and to produce juveniles for laboratory testing. Figure created using ArcMap 10.8.1 (https://www.esri.com/en-us/arcgis/products/arcgis-desktop/overview).

Gravid females were collected from two sites in the Devils River (Grass Patch and Ruthies; Fig. 1) in May 2019. Following collection, mussels were transported to the Texas A&M AgriLife Extension and Research Center in Dallas, TX in insulated coolers. Upon arrival at the laboratory, individual mussels were held at room temperature (20 ± 1 °C) in separate cups within a 20-gallon aquarium and fed twice daily with a 200 mL mixture of Shellfish Diet 1800 and Nanno 3600 Instant Algae (Reed Mariculture, San Jose, CA, USA) until testing. Females were held in separate cups at room temperature (20 ± 3 °C) within an aquarium until trials. Glochidia were extracted from females by flushing their gills with water using an 18-guage syringe.

### Thermal tolerance testing

Initial glochidial viability was assessed by exposing 10 subsamples of suspended glochidia of equal volume to a saturated NaCl solution. Glochidia were considered viable if they exhibited valve closure in response to the salt solution. When possible, subsamples with > 80% viability were used for thermal testing; however, since *P. popeii* is an endangered species and efforts were made to minimize disturbing the population, some individuals with < 80% glochidial viability were used. Corrections were made to account for these discrepancies. Subsamples were pooled together by location for three mussels from Ruthies (86 ± 5.85%) and two mussels from Grass Patch (56 ± 1.78%). Untested glochidia were used to produce juveniles by infecting three presumptive laboratory host fish, *Cyprinella lutrensis* (Leuciscidae; red shiner), *Gambusia affinis* (Poecilidae; western mosquitofish), and *Campostoma anomalum* (Leuciscidae; central stoneroller), following methods described by Johnson et al.^39^. Juveniles from Grass Patch were not tested due to issues with initial glochidial viability.

**Figure 2:**
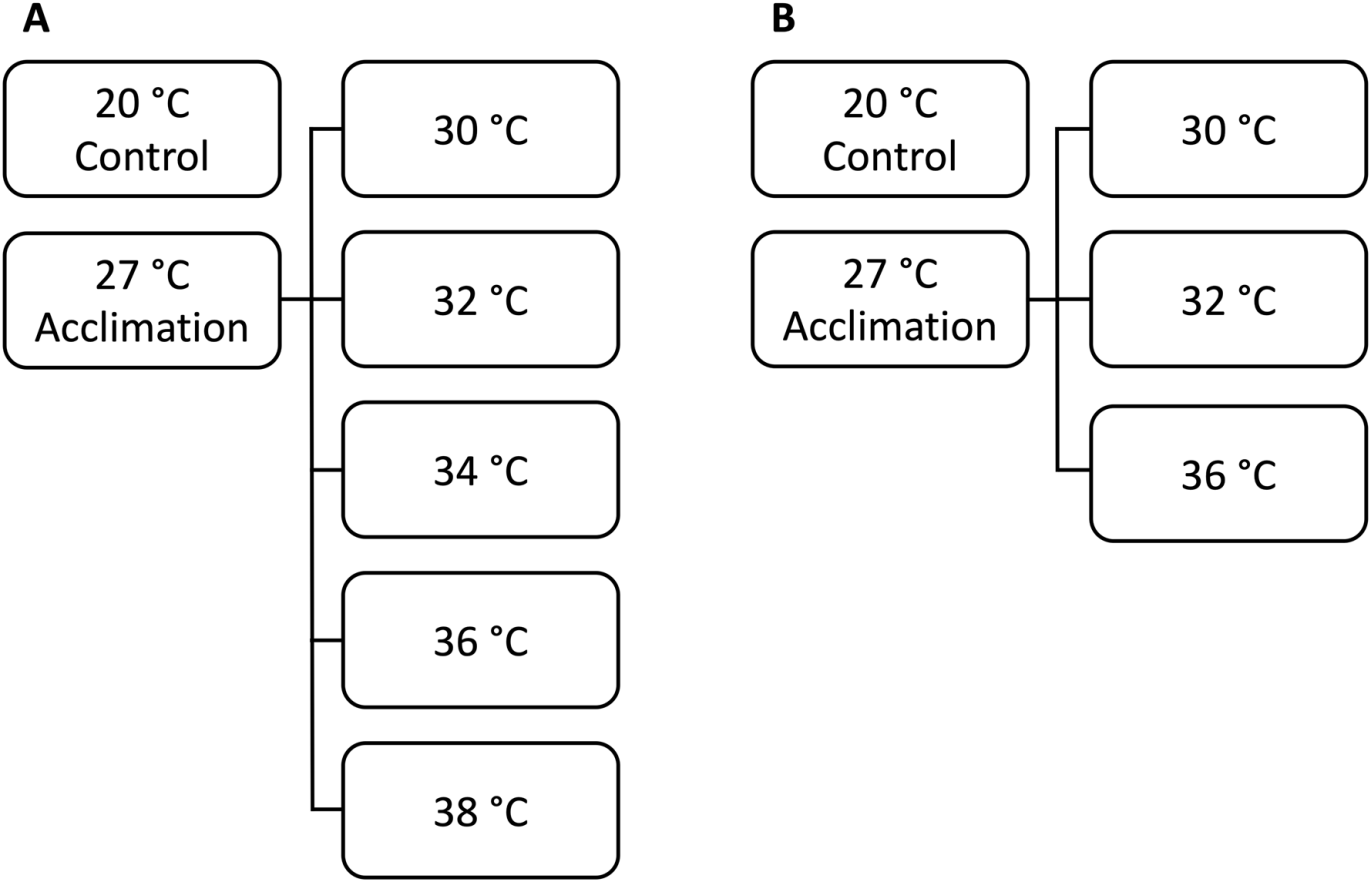
Experimental design following Khan et al.^24^ showing acclimation and experimental temperatures plus a non-acclimated control for (**A**) glochidia and (**B**) juveniles.

The upper thermal limits (LT05 and LT50) of glochidia and juveniles were estimated by adapting methods presented in Khan et al.^24,40^ Specifically, glochidia were acclimated to 27 °C from room temperature (20 ± 1 °C) by increments of 1 °C per hour in a refrigerated incubator. Once 27 °C was reached, glochidia were held at this temperature for two hours prior to testing as per ASTM International guidelines^41^. Tests were conducted by placing approximately 250 glochidia in a non-aerated 100 mL beaker filled with 80 mL reconstituted hard water^41^. Each beaker was then held in a fiberglass water bath containing approximately 5 L reverse osmosis water and maintained at one of five experimental temperatures (30, 32, 34, 36, and 38 °C) plus a non-acclimated control (20 °C) for 24-h (Fig. 2). Each test temperature had three replicates. Temperatures within the baths were maintained using a 300 W titanium heater attached to a temperature controller (Aqua Logic, San Diego, CA, USA). After 12-h and 24-h, glochidial viability was reassessed by taking a subsample of approximately 50 glochidia from each of the three replicates for each test temperature and exposing them to a saturated NaCl solution. Valve closure was assessed under a dissecting microscope (SZ51, Olympus America, Center Valley, PA, USA).

Newly transformed juveniles from Ruthies were acclimated to 27 °C from room temperature by increments of 1 °C per hour in a refrigerated incubator. Once 27 °C was reached, juveniles were held at this temperature for 24-h prior to trials in accordance with ASTM guidelines^41^. Juveniles were tested at three experimental temperatures (30, 32, and 36 °C) plus a non-acclimated control (20 °C) for 96-h under non-aerated renewal conditions. Each test temperature had three replicates containing approximately 10 juveniles each. After the first 48-h, a 90% water change was conducted using reconstituted hard water. After 96-h, juvenile survival was determined by visually searching for foot movement outside the shell using an Olympus SZ51 dissecting microscope (Olympus America, Center Valley, PA, USA).

### Water temperature and flow measurements

Hobo water level loggers (Onset Computer Corporation, Bourne, MA, USA) were deployed at Grass Patch and Ruthies on the Devils River to record water temperature (Fig. 1). Loggers were housed in perforated PVC pipe, which was secured to a piece of rebar and anchored to the streambed. They were deployed from 24 August 2020 to 17 August 2021 and recorded water temperature at 30-min intervals. Additional Ruthies water temperature data from 20 October 2015 to 25 March 2018 were obtained from Caldwell et al.^35^ to temporally expand our dataset. Discharge data from 20 October 2015 to 30 June 2020 were obtained from the International Boundary and Water Commission (IBWC) gaging station 08449400 (Devils River at Pafford Crossing near Comstock)^42^, located 21.6 km from Grass Patch and 25.2 km from Ruthies (Fig. 1). Mean daily discharge from the gaging station was related to maximum daily water temperature from the Hobo water level loggers.

### Statistical analysis

Lethal temperatures resulting in 5% and 50% mortality (LT05 and LT50, respectively) were determined for glochidia and juveniles by fitting survival data to a logistic regression model (n = 3). Because initial glochidial viability was under 100%, survival values were adjusted following methods described in Wang et al.^43^ and Khan et al.^24^ by dividing treatment viability (assessed after 12-h or 24-h) by initial viability. Only LT50 was determined for juveniles since there were only three experimental temperatures, and therefore LT05 could not be reliably calculated. LT05 and LT50 were calculated based on mortality (1—viability) at each test temperature.

All statistics were performed in R version 3.4.1^44^. The drm() function in the ‘drc’ package (version 1.0-1) was used to estimate LT05/50. For each pair of LT05/50 thresholds, seven models were created: four-parameter log-logistic, three parameter log-logistic, two-parameter log-logistic, four-parameter Weibull type 1, three-parameter Weibull type 1, four-parameter Weibull type 2, and three-parameter Weibull type 2. The best-fit model for each threshold was selected using the mselect() function in the ‘drc’ package as well as comparison of standard error and 95% confidence intervals. Confidence interval ratio tests were performed to assess whether there were significant differences between different thresholds using the comped() function in the ‘drc’ package^45^. In these tests, the ratio of each pair of thresholds (LT05 or LT50) is compared with one. The variance of each LT estimate is used to construct a 95% confidence interval for the ratio. The null hypothesis that the population LTs are the same is rejected if the 95% CI does not contain one^45^.

### Host fish thermal tolerance review

A literature review was conducted for thermal tolerance data on the following presumptive host fish for *P. popeii* identified in the Black River, New Mexico^46^: *Cyprinella lutrensis* (Leuciscidae; red shiner), *Carpiodes carpio* (Catostomidae; river carpsucker), and *Moxostoma congestum* (Catostomidae; gray redhorse). Data for the following congeners were also included: *Cyprinella venusta* (Leuciscidae; blacktail shiner), *Carpiodes cyprinus* (Catostomidae; quillback), *Moxostoma anisurum* (Catostomidae; silver redhorse), *Moxostoma erythrurum* (Catostomidae; golden redhorse), and *Moxostoma robustum* (Catostomidae; shorthead redhorse). Three of these species (*C. venusta*, *C. carpio*, and *M. congestum*) occur in the Devils River^47–49^. One species (*C. lutrensis*), is not present within the Devils River but occurs in other parts of *P. popeii*’s range, while the remaining four species (*C. cyprinus*, *M. anisurum*, *M. erythrurum*, and *M. robustum*) do not occur within *P. popeii*’s range^50^.

Data were compiled for critical thermal maximum (CTMax), critical lethal maximum (CLMax), lethal thermal maximum (LTMax), and maximum weekly temperature tolerance (MWTT) for the nine aforementioned fishes. CTMax is a sub-lethal, acute test, and is used to determine the upper temperature at which an organism becomes disabled and can no longer escape lethal conditions^51–53^. CTMax is evaluated by exposing fish to rapidly increasing temperature and monitoring the following endpoints: loss of equilibrium (initial or final), loss of righting response, onset of spasms, and flaring opercula. LTMax follows the same methodology as CTMax but with death as the endpoint, so this method is used to determine the average temperature at which an individual dies due to overheating^52^. CLMax is a lethal, chronic test, used to determine the average temperature at which an individual is no longer able to survive gradual heating, which may be more representative of actual conditions experienced in the wild than LTMax^54,55^. CLMax is determined by exposing fish to gradually increasing temperatures, usually over several days, until death. MWTT is used to determine the maximum temperature suitable for a given species to live^56^. It is calculated by overlaying in situ water temperature data onto species distribution data. After reviewing host fish thermal tolerance, we related these values to in situ water temperature data from Grass Patch and Ruthies to determine whether water temperature could be limiting to *P. popeii*’s presumptive hosts in the Devils River.

### Ethical statement

Collection of adult mussels was covered under Texas Parks and Wildlife Scientific Collection Permit No. SPR-0511-142 and U.S. Fish and Wildlife Permit No. TE79165C-0. All mussel production and experimental protocols were approved by the Texas A&M University Institutional Animal Care and Use Committee and conducted in accordance with Animal Care and Use Protocol AUP No. 2019-016A. No thermal testing was conducted on vertebrates. All methods are reported in accordance with ARRIVE guidelines.

### Consent to participate

All authors agreed on the submission.

## Results

### Lethal temperature exposures

**Figure 3:**
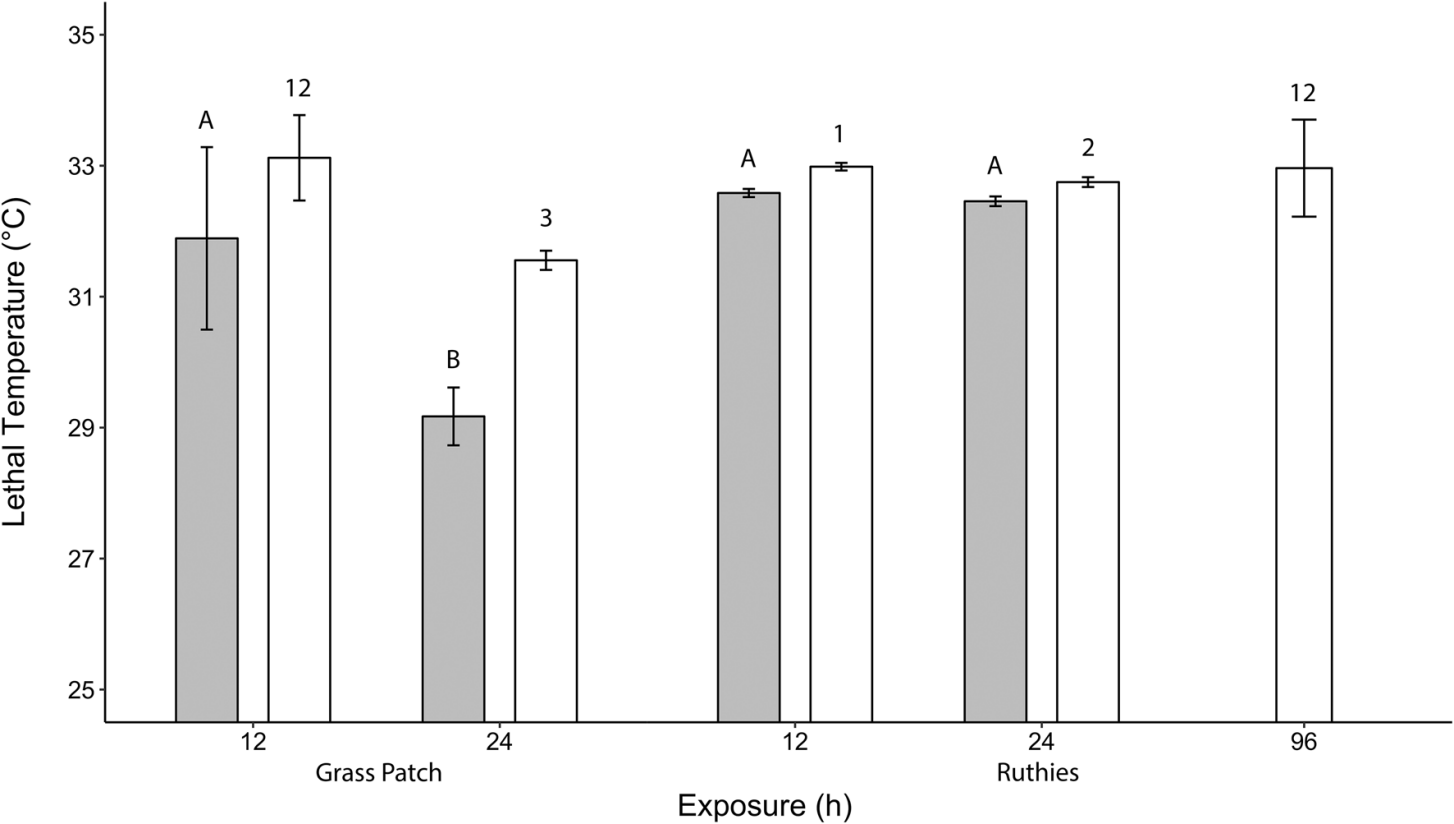
Comparison of lethal temperatures (LT) for 5% (gray bars) and 50% (white bars) of *P. popeii* glochidia (12-h and 24-h) and juveniles (96-h) from two sites in the Devils River. Error bars indicate 95% confidence intervals. LT05 values annotated with the same letters are not significantly different and LT50 values annotated with the same numbers are not significantly different based on the confidence interval ratio test (*p* < 0.05).

Twelve-hour LT05 values for glochidia at Grass Patch and Ruthies were 31.9 °C (95% CI 30.5–33.3 °C) and 32.6 °C (95% CI 32.5–32.6 °C), which were not significantly different based on a confidence ratio test (Fig. 3; Table 1). Twenty-four-hour LT05 values for glochidia at Grass Patch and Ruthies were 29.9 °C (95% CI 28.7–29.6 °C) and 32.5 °C (95% CI 32.4–32.5 °C), which were significantly different based on a confidence interval ratio test (Fig. 3; Table 1). Twelve-hour and 24-h LT05 for Grass Patch glochidia were significantly different based on a confidence interval ratio test (Fig. 3); likewise, 12-h and 24-h LT05 for Ruthies glochidia were significantly different (Fig. 3). Twelve-hour LT50 values for glochidia at Grass Patch and Ruthies were 33.1 °C (95% CI 32.5–33.8 °C) and 33.0 °C (95% CI 32.9–33.0 °C), which were not significantly different based on a confidence interval ratio test (Fig. 3; Table 1). Twenty-four-hour LT50 values for glochidia at Grass Patch and Ruthies were 31.6 °C (95% CI 31.4–31.7 °C) and 32.7 °C (95% CI 32.7–32.8 °C), which were significantly different based on a confidence interval ratio test (Fig. 3; Table 1). Twelve-hour and 24-h LT50 values for Grass Patch glochidia were significantly different based on a confidence interval ratio test (Fig. 3); likewise, 12-h and 24-h LT50 for Ruthies glochidia were significantly different (Fig. 3). For 96-h juveniles at Ruthies, LT50 was 33.0 °C (95% CI 32.2–33.7 °C), which was not significantly different from LT50 for glochidia at Ruthies based on a confidence interval ratio test (Fig. 3; Table 1). The LT05 for 96-h juveniles from Ruthies could not be accurately calculated due to the small number of treatments. Juveniles from Grass Patch were not tested due to issues with initial glochidial viability.

**Table 1:** Laboratory derived lethal temperatures for 5% (LT05) and 50% (LT50) of *Popenaias popeii* glochidia and juveniles from two Devils River populations at 12, 24, and 96-h timepoints. Mean ± SE (95% CI), N = 3.

Population	12-h glochidia		24-h glochidia		96-h juvenile
	LT05	LT50	LT05	LT50	LT50
Grass Patch	31.9 ± 0.6331 (30.5–33.3)	33.1 ± 0.2963 (32.5–33.8)	29.2 ± 0.2006 (28.7–29.6)	31.6 ± 0.0667 (31.4–31.7)	–
Ruthies	32.6 ± 0.02886 (32.5–32.6)	33.0 ± 0.0264 (32.9–33.0)	32.5 ± 0.0337 (32.4–32.5)	32.7 ± 0.0340 (32.7–32.8)	33.0 ± 0.3133 (32.2–33.7)

### Thermal exceedances

**Figure 4:**
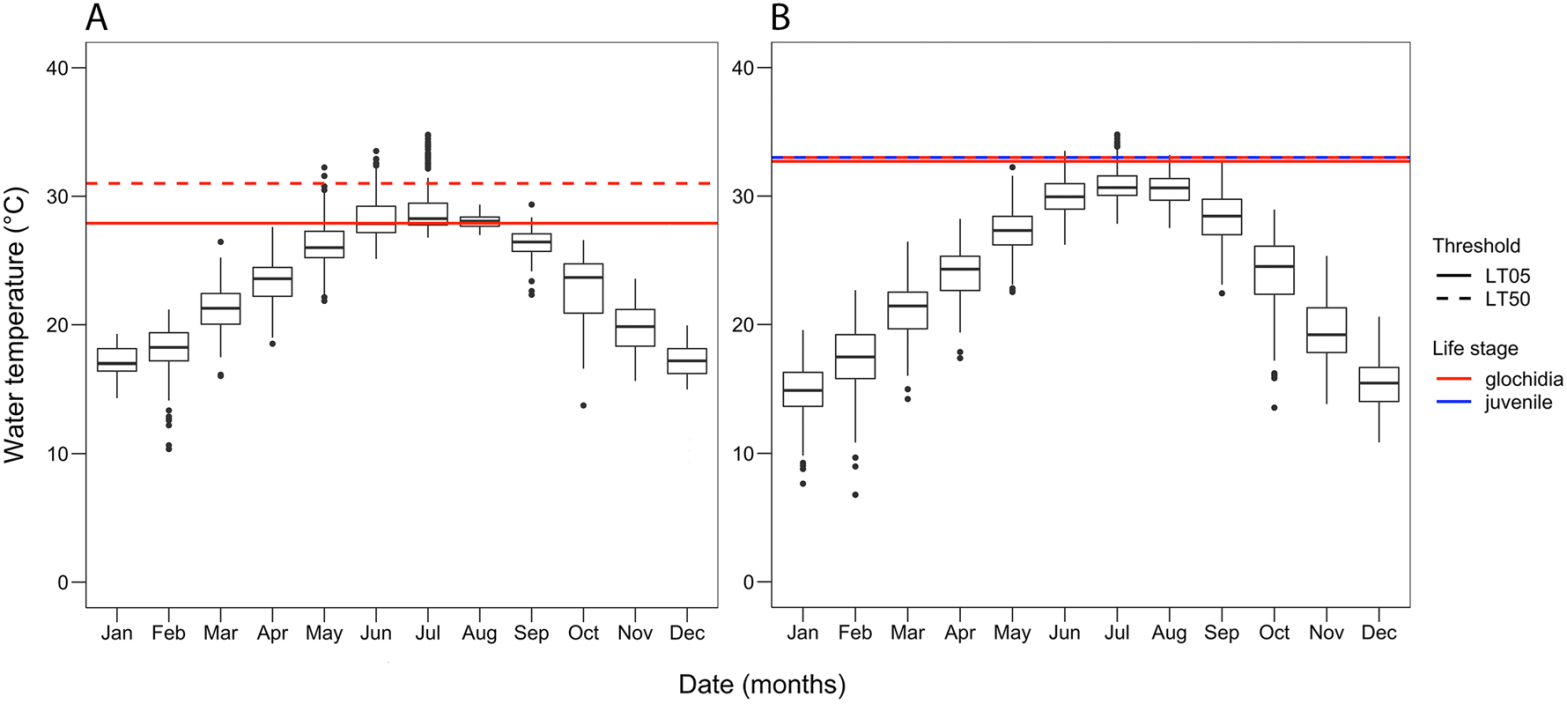
Water temperature and thermal tolerance data for two subpopulations of *P. popeii* in the Devils River. Red lines = glochidia, blue lines = juveniles, solid lines = LT05, and dashed lines = LT50. (**A**) Comparison of maximum daily water temperature at Grass Patch from March 2018 to August 2020 and laboratory derived thermal tolerance of Grass Patch *P. popeii* glochidia (24-h). (**B**) Comparison of maximum daily water temperature at Ruthies from October 2015 to August 2020 and laboratory derived thermal tolerance of Ruthies *P. popeii* for both glochidia (24-h) and juveniles (96-h).

**Figure 5:**
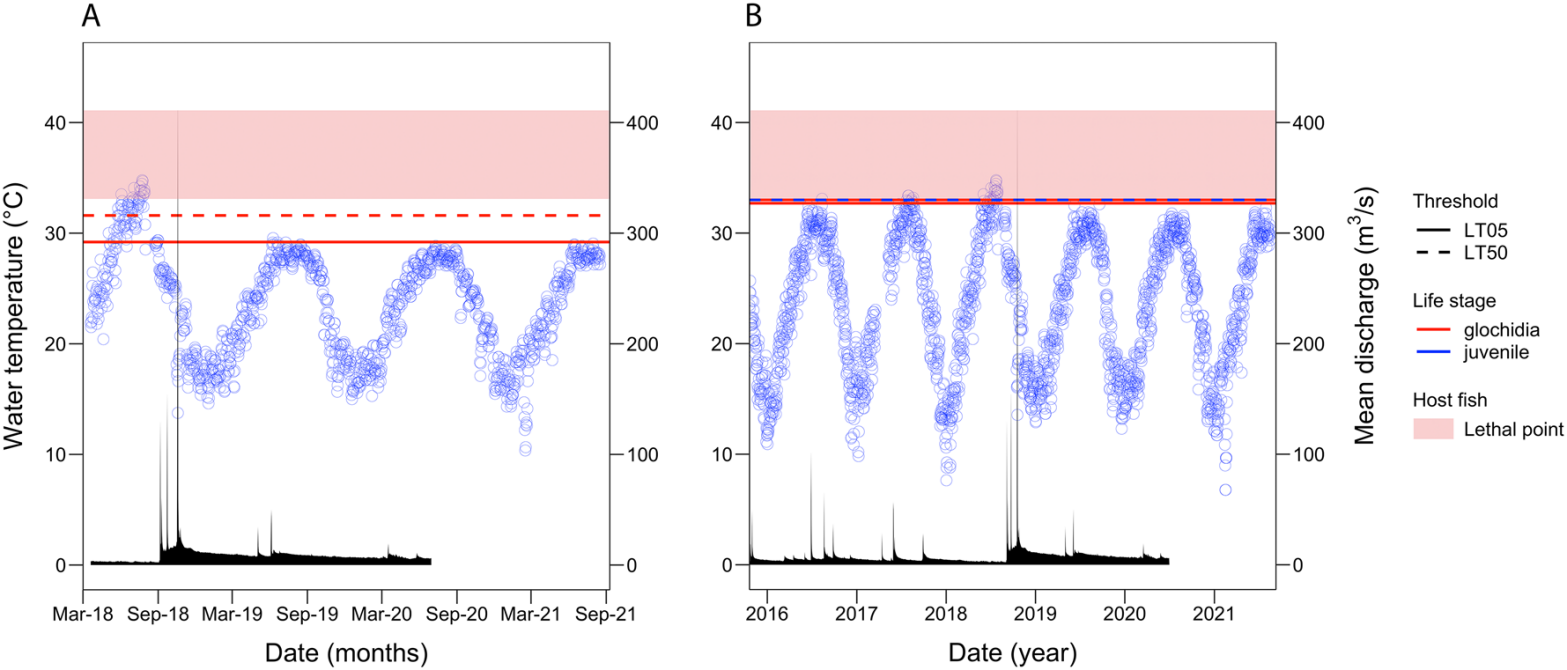
Water temperature and discharge data from two sites in the Devils River overlaid with thermal tolerance data for *P. popeii* and its host fish. Blue circles = maximum daily water temperature, black polygon = mean daily discharge, red lines = thresholds for glochidia, blue lines = thresholds for juveniles, solid lines = LT05, dashed lines = LT50, and pink rectangle = host fish lethal tolerance range. (**A**) Comparison of maximum daily water temperature at Grass Patch from March 2018 to August 2020 to laboratory derived thermal tolerance of Grass Patch *P. popeii* and its presumptive host fish. (**B**) Comparison of maximum daily water temperature at Ruthies from October 2015 to August 2020 to laboratory derived thermal tolerance of Ruthies *P. popeii* and its presumptive host fish.

At Grass Patch, maximum daily water temperature from March 2018 to August 2021 ranged from 10.4 to 34.8 °C and was lowest in February 2021 and highest in July 2018 (Fig. 4A). The highest absolute water temperature recorded was 34.8 °C and occurred 24 July 2018, when mean daily discharge was 2.8 m^3^ s^–1^. Mean daily discharge from March 2018 to June 2020 ranged from 2.4 to 411 m^3^ s^–1^, reaching its lowest point in September 2018 and highest point in October 2018 (Fig. 5A). There were six exceedances of the 24-h LT05 threshold by maximum daily water temperature, which averaged 20 days (range: 1–45 days, total: 75 days; average mean daily discharge: 3.1 m^3^ s^–1^). Five exceedances occurred in 2018 and one occurred in 2019 (Table 2). The longest event occurred from 17 June to 31 July 2018, which had an average mean daily discharge of 2.9 m^3^ s^–1^. The LT50 threshold for Grass Patch glochidia was exceeded six times for an average of 6.8 days (range: 1–20 days, total: 41 days; average mean daily discharge: 2.9 m^3^ s^–1^), all of which occurred in 2018 (Table 2). The longest of these exceedance events occurred from 11 to 30 July 2018, when mean daily discharge averaged 2.8 m^3^ s^–1^.

**Table 2:** Water temperature and discharge data for temperature exceedance events for the LT05 and LT50 thresholds (24-h glochidia and 96-h juvenile) for two subpopulations of *Popenaias popeii* in the Devils River.

Population	Life stage	Threshold	Maximum daily temp. (°C)	Mean daily discharge (cm^3^ s^−1^)
Grass Patch	Glochidia	LT05	29.4–34.8	2.4–10.2
		LT50	31.9–34.8	2.4–3.6
Ruthies	Glochidia	LT05	32.5–34.8	2.4–8.3
		LT50	33.0–34.8	2.4–5.2
	Juvenile	LT50	33.0–34.8	2.4–5.2

Ranges are presented for maximum daily temperature and mean daily discharge.

At Ruthies, maximum daily water temperature from October 2015 to August 2021 ranged from 6.8 to 34.8 °C and was lowest in February 2016 and highest in July 2018 (Fig. 4B). The highest absolute water temperature recorded at Ruthies was 34.8 °C and occurred on 24 July 2018, when mean daily discharge was 2.8 m^3^ s^–1^. Mean daily discharge from October 2015 to August 2021 ranged from 2.4 to 411 m^3^ s^–1^ and was lowest in November 2018 and highest in October 2018 (Fig. 5B). Maximum daily water temperature exceeded the 24-h LT05 threshold for glochidia 16 times for an average of 2.9 days (range: 1–13 days, total: 46 days; average mean daily discharge: 3.5 m^3^ s^–1^). Nine exceedances occurred in 2018 and four occurred in 2017 (Table 2). The longest exceedance occurred from 14 to 26 July 2018 when mean daily discharge averaged 2.8 m^3^ s^–1^ (Fig. 4B). Likewise, maximum daily water temperature exceeded the 24-h LT50 threshold for glochidia 14 times for an average of 2.2 days (range: 1–6 days, total: 31 days; average mean daily discharge: 3.2 m^3^ s^–1^). Three exceedances occurred in 2017 and one occurred in 2016 (Table 2). The longest exceedance occurred from 15 to 20 July 2018 when mean daily discharge averaged 2.8 m^3^ s^–1^. The 96-h LT50 for Ruthies juveniles was exceeded 11 times for an average of 1.9 days (range: 1–5 days; total: 21 days; average mean daily discharge: 3.2 m^3^ s^–1^) (Fig. 4B; Table 2). The longest exceedance occurred from 22 to 26 July when mean daily discharge averaged 2.7 m^3^ s^–1^.

### Host fish thermal tolerance review

A total of 15 papers were identified containing sublethal and lethal thresholds for *P. popeii*’s presumptive hosts (*C. carpio*, *C. lutrensis*, and *M. congestum*) and their congeners (*C. cyprinus*, *C. venusta*, *M. anisurum*, *M. erythrurum*, *M. macrolepidotum*, and *M. robustum*) as previously explained^51,53–66^.

CTMax was the most commonly conducted test, with data available for *C. carpio*, *C. cyprinus*, *C. lutrensis*, and *M. robustum*. Values ranged from 30.7 to 40.6 °C for all studies included in our review (see supplementary Table S1 online). CTMax values for host fish were generally higher than LT05/50 values found for mussels acclimated to 27 °C in our study. *Carpiodes carpio*, when acclimated to 23.7 °C, had a CTMax of 36.6 °C^64^. Similarly, *C. cyprinus* had a CTMax of 38.8  C when acclimated to 24.0 °C^62^. *Cyprinella lutrensis* had the greatest number of CTMax tests over the widest range of acclimation temperatures, with a value of 38.1 °C when acclimated to 26 °C (see supplementary Table S1 online)^63^. *Moxostoma robustum* had a CTMax of 34.9 °C when acclimated to 20 °C and a value of 37.2 °C when acclimated to 30 °C^65^.

Several studies reported LTMax for *C. carpio*, *C. lutrensis*, *C. venusta*, and *M. erythrurum*, which ranged from 35.1 to 40.9 °C (see supplementary Table S1 online). *Carpiodes carpio* had an LTMax of 38.0 °C when acclimated to 23.7 °C^64^. *Cyprinella lutrensis* had an LTMax of 39.5 °C when acclimated to 25 °C and 40.9 °C when acclimated to 30 °C^53^. *Moxostoma macrolepidotum* had an LTMax of 35.1 °C when acclimated to 20.6–23.8 °C^54^. LTMax values for these three fishes were higher than the LT05/50 values for *P. popeii* acclimated to 27 °C in our study.

CLMax was reported for *C. venusta* and *M. macrolepidotum*. *Cyprinella venusta* had a CLMax of 39.0 °C when acclimated to 20 °C, which is higher than mussel LT05/50 in our study, and *M. macrolepidotum* had a CLMax of 33.3 °C when acclimated to 10.6–23.8 °C, which is very similar to LT05/50 values for mussels acclimated to 27 °C in our study^54,55^. One study reported MWTT for various freshwater fishes using water temperature data and reported a value of 29.6 °C for *M. erythrurum* and *M. macrolipidotum* and 32.1 °C for *C. carpio*^56^, which was similar to mussel thermal tolerance found in our study. However, it should be noted that the MWTT methodology is not lethal.

## Discussion

We successfully estimated the upper thermal limits (LT05 and LT50) of *P. popeii* glochidia and newly transformed juveniles from the Devils River. We also identified similar thermal performance endpoints based on a literature review of *P. popeii*’s presumptive host fish and their congeners. Comparing these thermal limits with in situ water temperature data, we found few temperature exceedances, indicating *P. popeii* and its presumptive hosts may not currently be experiencing thermal stress. This may explain why the Devils River continues to be a stronghold for *P. popeii* and other rare aquatic species. The few exceedances we did observe occurred during the summer months (June–August) and were mostly in 2018, a period of low precipitation (USGS gage 08449100 Dolan Ck abv Devils River nr Comstock, TX)^67^. It is well known that changes to discharge and water volume in a river affect the rate at which it heats and cools^2^, and thus warmer stream temperatures during summer months are not unexpected. Because of this, summer months have been described as an environmental flow bottleneck for mussels and fish because they overlap with key aspects of population performance (growth, survivorship, and reproduction)^68^. This appears to be the case for the Devils River, and so conservation managers focused on protecting mussels and fish in this system should pay close attention to river discharge during the summer period.

The Devils River is also an anomaly to this pattern, not because it doesn’t experience low flows during summer months, but because mussel thermal tolerance exceedance during this time is reduced relative to regulated streams. For example, Khan et al.^40^ demonstrated for adults of the unionid *Fusconaia mitchelli* (false spike) in the Guadalupe River, a heavily regulated spring-fed system, that mean daily water temperature exceeded 96-h acute LT05 seven times during the spring reproductive period and 12 times during the summer period. Goldsmith et al.^25^ found thermal tolerances for glochidia and juveniles of *Lampsilis bracteata* (Texas fatmucket), in the San Saba River were exceeded on average 4.5 times for up to 75 days (24-h LT05) and 12.5 times for up to 33 days (96-h LT05) during the summer periods from 2017 to 2019. The San Saba River is also a spring-fed system, heavily used for agricultural and commercial activities. Temperature exceedances like the ones reported by Khan et al.^40^ and Goldsmith et al.^25^ can negatively impact mussel survival, growth, and reproduction, which affects long-term viability^69,70^.

Karst springs are important features in semi-arid to arid environments because they provide a permanent water source, which can help mitigate poor water quality and declines in streamflow^49^. In the Devils River, it is estimated that spring inputs contribute approximately 40% of total discharge, resulting in baseflows characterized by low variation in conductivity, pH, and dissolved oxygen, and moderate seasonal variation in water temperature^35^. This consistency in water quality and quantity is a likely explanation, at least in part, for the infrequency of mussel thermal tolerance exceedances in the Devils River. Randklev et al.^38^ made a similar observation for *P. popeii* inhabiting the Lower Canyons of the Rio Grande. The authors noted reduced flows and impaired water quality from the Rio Conchos were offset by groundwater from springs and adjacent aquifers beginning near Big Bend National Park, which culminated in the Lower Canyons^38^. Occupancy for *P. popeii* in this reach followed this same pattern such that occurrence was reduced near the confluence with the Rio Conchos and maximized in the Lower Canyons^38^. Our findings, in conjunction with those from the Lower Canyons, illustrate the importance of spring inputs in maintaining flow and improving water quality for mussels.

We also found that presumptive host fish lethal temperature tolerance was higher than that of *P. popeii*, suggesting mussels may be more sensitive to hydrologic alterations than their host fish. Spooner et al.^71^ made a similar observation, noting mussel richness is more closely associated with discharge compared to their hosts. In our study, fish lethal tolerance ranged from 33.3 to 40.9 °C, whereas LT05 and LT50 thresholds for *P. popeii* glochidia and juveniles ranged from 29.2 to 33.0 °C (Fig. 4). The exact reason for this difference is unclear, but could be due to *P. popeii*’s sedentary nature compared to its presumptive host fish, which have a greater ability to find more favorable conditions. While there is evidence that some unionids may track receding water during droughts or burrow to avoid desiccation^72^, this has not been demonstrated experimentally for temperature. Archambault et al.^73,74^ found that sublethal thermal stress significantly reduced burrowing behavior in juveniles of several *Lampsilis* species. However, it is possible that mussels could seek thermal refuge by burrowing or moving short distances across the streambed. In addition to being more mobile, host fish may also have greater thermoregulatory ability than mussels. However, it is important to note that thermal tolerance values reviewed for host fish (CTMax, CLMax, LTMax, and MWTT) are based on different methodologies and acclimation temperatures than mussel LT, and it is therefore difficult to make exact comparisons between the endpoints.

### Conservation implications

Our study provides a useful approach to evaluating the thermal tolerance of an aquatic ectotherm and placing it within an ecological context. It also highlights the importance of considering the response of interspecific relationships to environmental change. For *P. popeii* in the Devils River, future studies could build upon our findings by determining thermal tolerance using diel swings in water temperature. This information could be important because maximum daily water temperatures occur only for a short time each day, and mussels may have the opportunity to recover as temperature decreases during the diel cycle. For example, Martin^75^ conducted LT50 trails for freshwater mussels in which water temperatures were increased to different peak temperatures to mimic a natural diel cycle. When compared to other 7-d juvenile LT50 studies using static temperatures, Martin obtained values that were at least 3 °C higher^75^. Thus, the use of diel swings could provide more accurate thermal tolerance estimates. Additionally, the impact of temperature on the mussel-host relationship can be more directly assessed by studying how elevated temperature impacts the attachment and metamorphosis success of glochidia on fish.

In this study, overlaying laboratory derived LT05 and LT50 estimates for glochidia and newly transformed juveniles against in situ water temperature data shows greatest thermal stress from June through August. Discharge during exceedances for this period ranged from 2.4 to 10.2 cm^3^ s^−1^ across both Grass Patch and Ruthies (Table 2). Thus, flows below these values could lead to further thermal stress and, depending on their frequency and duration, negatively impact mussel population persistence. This window could be further improved with additional information on spawning and brooding period, host fish behavior (i.e., glochidia-host fish contact), and growth rates. Additionally, information on how this window shifts between wet and dry years could provide insight into how mussels cope with changes in flow and temperature. This type of information would allow natural resource managers to tailor recommendations to different flow periods.

As climate change intensifies and human demand for fresh water grows, it is critical to balance the water needs of humans and wildlife. For threatened and endangered species in the United States, USFWS typically designates “critical habitat” and state resource managers may also create recovery plans. In the case of *P. popeii*, the Federal Recovery Implementation Plan identifies flow management to support population performance as a key action for sustaining this species^76^. The state of New Mexico also signed a Candidate Conservation Agreement with Assurances for *P. popeii* in the Black River, which stipulates a minimum flow requirement to protect its habitat^77^. However, this flow requirement can be refined by incorporating thermal tolerance data from mussels and their host fish. As demonstrated in our study, thermal exceedances can be linked to discharge levels, which can be used to create more accurate flow targets for a variety of aquatic organisms. This approach serves as a practical framework for both researchers and natural resource managers to assess the hydrological needs of aquatic organisms, including parasite-host relationships.

## Supplementary Information

Supplementary Information.

## Data Availability

The datasets generated during and/or analyzed during the current study are available from the corresponding author on reasonable request.
