# Supplementary material for: Thermal tolerances of Popenaias popeii (Texas hornshell) and its host fish from the Rio Grande Basin, Texas
Source: Sci Rep. 2023 Mar 21;13:4603. doi: 10.1038/s41598-023-29460-9 (PMC10030872; doi:10.1038/s41598-023-29460-9)
Supplement: Supplementary file 1 — Supplementary Information. [file 41598_2023_29460_MOESM1_ESM.docx]

**Table S1.** Thermal maxima data for three freshwater fishes presumed to be ecological hosts for the glochidia of the freshwater mussel *Popenaias popeii*. Each entry includes life stage and size (TL = total length, SL = standard length), ambient temperature, pretest acclimation temperature, acclimation time, thermal test performed (CTMax = critical temperature maximum, LTMax = lethal temperature maximum, CLMax = chronic lethal maximum, MWTT = maximum weekly temperature tolerance), reported test endpoint ((I/F)LOE = (initial/final) loss of equilibrium, LRR = loss of righting response, OS = onset of spasms, FO = flaring opercula, D = death) and rate of temperature increase. Values estimated from plots are denoted by superscript a. SDs calculated from variance, SE, or 95% CI are denoted by superscript b. Graph estimates obtained from Beitinger et al. (2000) are denoted by superscript c. SDs calculated based on the lowest possible value of n are denoted by superscript *. Lethal temperature data used in Fig. 5 are in bolded text.

| Species | Location | Life stage / size | T_ambient_ | T_acclimation_ °C | Acclimation time | Test | Endpoint | ∆ T | Thermal tolerance, °C | | | Reference |
| --- | --- | --- | --- | --- | --- | --- | --- | --- | --- | --- | --- | --- |
|  |  |  |  |  |  |  |  |  | x̅ | SD | n |  |
| *Carpiodes carpio* | Platte River, NE | — | x̅ = 23.7 | 23.7 | ≥ 14 d | CTMax | LOE | 1 °C min^-1^ | 36.3 | Range: 30.2–37.9 | 10 | 64 |
|  | **Platte River, NE** | **—** | **x̅ = 23.7** | **23.7** | **≥ 14 d** | **LTMax** | **D** | **1 °C min^-1^** | **38.0** | **Range: 33.1–39.4** | **10** | **64** |
|  | Across the US | — | “warm water” | — | — | MWTT | — | — | 32.1 | — | — | 56 |
| *Carpiodes cyprinus* | Indian Creek, CO | — | — | 24.0 | ≤ 3 h | CTMax | LRR | 0.5–0.8 °C min^-1^ | 38.8 | 0.8 | 6 | 62 |
| *Cyprinella lutrensis* | S.C. OK (streams & reservoirs) | — | — | 10 ± 1 | ≥ 2 w | CTMax | LRR | 1 °C min^-1^ | 30.70 | 1.01^b^ | 20 | 51 |
|  | S.C. OK (streams & reservoirs) | — | — | 10 ± 1 | ≥ 2 w | CTMax | OS | 1 °C min^-1^ | 34.04 | 0.894^b^ | 20 | 51 |
|  | Walnut Creek, OK | — | (Fall) | 15 | ≥ 1 w | CTMax (initial) | OS | 1 °C min^-1^ | 32.0^d^ | 1.04^c^ | 82 | 58 |
|  | Walnut Creek, OK | — | (Fall) | 15 | ≥ 1 w | CTMax  (1 h) | OS | 1 °C min^-1^ | 32.8^a^ | 1.26^a,b^ | 13 | 58 |
|  | Walnut Creek, OK | — | (Fall) | 15 | ≥ 1 w | CTMax  (2 h) | OS | 1 °C min^-1^ | 32.5^a^ | 1.08^a,b^ | 13 | 58 |
|  | Walnut Creek, OK | — | (Fall) | 15 | ≥ 1 w | CTMax  (4 h) | OS | 1 °C min^-1^ | 32.6^a^ | 0.95^a,b^ | 10 | 58 |
|  | Walnut Creek, OK | — | (Winter) | 15 | ≥ 1 w | CTMax (initial) | OS | 1 °C min^-1^ | 31.4^a^ | 0.47^a,b,*^ | ≥ 10 | 58 |
|  | Walnut Creek, OK | — | (Winter) | 15 | ≥ 1 w | CTMax  (1 h) | OS | 1 °C min^-1^ | 31.9^a^ | 1.11^a,b,*^ | ≥ 10 | 58 |
|  | Walnut Creek, OK | — | (Spring) | 15 | ≥ 1 w | CTMax (initial) | OS | 1 °C min^-1^ | 33.4^a^ | 0.47^a,b,*^ | ≥ 10 | 58 |

Table S1. (Continued)

| Species | Location | Life stage / size | T_ambient_ | T_acclimation_ °C | Acclimation time | Test | Endpoint | ∆ T | Thermal tolerance, °C | | | Reference |
| --- | --- | --- | --- | --- | --- | --- | --- | --- | --- | --- | --- | --- |
|  |  |  |  |  |  |  |  |  | x̅ | SD | n |  |
|  | Walnut Creek, OK | — | (Spring) | 15 | ≥ 1 w | CTMax  (1 h) | OS | 1 °C min^-1^ | 34.9^a^ | 0.47^a,b,*^ | ≥ 10 | 58 |
|  | Denton County, TX (11 streams) | TL: 33.3–34.5 mm | — | 22 ± 0.1 | 2 w | CTMax | LRR | 0.3 °C min^-1^ | 36.18 | 0.49 | 12 | 59 |
|  | Palette River, ME (control fish) | SL: 40–60 mm | — | 23 | 14 d | CTMax | LOE | 1 °C min^-1^ | 36.5 | 1.5^b^ | 9 | 66 |
|  | Palette River, ME (control fish) | SL: 40–60 mm | — | 30 | 14 d | CTMax | LOE | 1 °C min^-1^ | 38.3 | 1.5 | 9 | 66 |
|  | Brazos River, TX (position from dam) |  |  |  |  |  |  |  |  |  |  |  |
|  | Upstream | 0.5–1.2 g | — | 25 | 4 w | CTMax | LRR | 0.3 °C min^-1^ | 37.99 | 0.63^b^ | — | 60 |
|  | 1 km | 0.5–1.2 g | — | 25 | 4 w | CTMax | LRR | 0.3 °C min^-1^ | 36.47 | 0.89^b^ | — | 60 |
|  | 30 km | 0.5–1.2 g | — | 25 | 4 w | CTMax | LRR | 0.3 °C min^-1^ | 37.15 | 0.94^b^ | — | 60 |
|  | 57 km | 0.5–1.2 g | — | 25 | 4 w | CTMax | LRR | 0.3 °C min^-1^ | 37.80 | 0.45^b^ | — | 60 |
|  | 121 km | 0.5–1.2 g | — | 25 | 4 w | CTMax | LRR | 0.3 °C min^-1^ | 37.81 | 0.41^b^ | — | 60 |
|  | South Canadian River | SL: 30–45 mm | — | 25 ± 1 | ≥ 2 w | CTMax | OS | 1 °C min^-1^ | 38.99 | 0.160 | 10 | 57 |
|  | AZ streams | TL: 30–70 mm | “warm water” | 25 | 14 d | CTMax | ILOE | 0.3 °C min^-1^ | 37.6 | 0.5^b*^ | ≥12 | 53 |
|  | AZ streams | TL: 30–70 mm | “warm water” | 25 | 14 d | CTMax | FLOE | 0.3 °C min^-1^ | 37.6 | 0.5^b*^ | ≥12 | 53 |
|  | AZ streams | TL: 30–70 mm | “warm water” | 25 | 14 d | CTMax | FO | 0.3 °C min^-1^ | 39.3 | 0.1^b*^ | ≥ 12 | 53 |
|  | **AZ streams** | **TL: 30–70 mm** | **“warm water”** | **25** | **14 d** | **LTMax** | **D** | **0.3 °C min^-1^** | **39.5** | **0.3^b*^** | **≥12** | **53** |
|  | AZ streams | TL: 30–70 mm | “warm water” | 30 | 14 d | CTMax | ILOE | 0.3 °C min^-1^ | 39.7 | 0.3^b*^ | ≥12 | 53 |
|  | AZ streams | TL: 30–70 mm | “warm water” | 30 | 14 d | CTMax | FLOE | 0.3 °C min^-1^ | 40.4 | 0.5^b*^ | ≥12 | 53 |
|  | AZ streams | TL: 30–70 mm | “warm water” | 30 | 14 d | CTMax | FO | 0.3 °C min^-1^ | 40.6 | 0.2^b*^ | ≥12 | 53 |

Table S1. (Continued)

| Species | Location | Life stage / size | T_ambient_ | T_acclimation_ °C | Acclimation time | Test | Endpoint | ∆ T | Thermal tolerance, °C | | | Reference |
| --- | --- | --- | --- | --- | --- | --- | --- | --- | --- | --- | --- | --- |
|  |  |  |  |  |  |  |  |  | x̅ | SD | n |  |
|  | Solomons River, KS | Adult | x̅: 25.5^a^, max: 33^a^ | 21 | 10– 14 d | CTMax | LOE | 1 °C min^-1^ | 36.30 | 0.36^b^ | 19 | 61 |
|  | Saline River, KS | Adult | max: 28^a^ | 21 | 10– 14 d | CTMax | LOE | 1 °C min^-1^ | 36.21 | 0.44^b^ | 15 | 61 |
|  | Smokey Hill River, KS | Adult | x̅: 28^a^, max: 32^a^ | 21 | 10– 14 d | CTMax | LOE | 1 °C min^-1^ | 36.21 | 0.53^b^ | 15 | 61 |
|  | Arkansas River, KS | Adult | x̅: 27^a^, max: 33^a^ | 21 | 10– 14 d | CTMax | LOE | 1 °C min^-1^ | 36.23 | 0.46^b^ | 15 | 61 |
|  | Rattlesnake Creek, KS | Adult | — | 21 | 10– 14 d | CTMax | LOE | 1 °C min^-1^ | 36.29 | 0.39^b^ | 15 | 61 |
|  | Ninnescah River, KS | Adult | — | 21 | 10– 14 d | CTMax | LOE | 1 °C min^-1^ | 36.15 | 0.35^b^ | 15 | 61 |
|  | Chikaski River, KS | Adult | — | 21 | 10– 14 d | CTMax | LOE | 1 °C min^-1^ | 35.92 | 0.35^b^ | 15 | 61 |
|  | Salt Fork River, OK | Adult | x̅: 30^a^, max: 35^a^ | 21 | 10– 14 d | CTMax | LOE | 1 °C min^-1^ | 36.24 | 0.35^b^ | 15 | 61 |
|  | North Canadian River, OK | Adult | x̅: 29^a^, max: 35^a^ | 21 | 10– 14 d | CTMax | LOE | 1 °C min^-1^ | 35.90 | 0.42^b^ | 15 | 61 |
|  | South Canadian River, OK | Adult | x̅: 32^a^, max: 36^a^ | 21 | 10– 14 d | CTMax | LOE | 1 °C min^-1^ | 36.18 | 0.37^b^ | 15 | 61 |
|  | Washita River, OK | Adult | x̅: 31^a^, max: 35^a^ | 21 | 10– 14 d | CTMax | LOE | 1 °C min^-1^ | 36.27 | 0.54^b^ | 15 | 61 |
|  | Red River, OK-TX | Adult | x̅: 32.^a^, max: 37.5^a^ | 21 | 10– 14 d | CTMax | LOE | 1 °C min^-1^ | 36.23 | 0.69^b^ | 15 | 61 |
|  | Trinity River, TX | Adult | x̅: 31^a^, max: 37^a^ | 21 | 10– 14 d | CTMax | LOE | 1 °C min^-1^ | 36.10 | 0.69^b^ | 15 | 61 |
|  | Brazos River, TX | Adult | x̅: 32^a^, max: 35^a^ | 21 | 10– 14 d | CTMax | LOE | 1 °C min^-1^ | 36.24 | 0.77^b^ | 15 | 61 |
|  | Paluxy River, TX | Adult | — | 21 | 10– 14 d | CTMax | LOE | 1 °C min^-1^ | 35.96 | 0.44^b^ | 15 | 61 |
|  | Leon River, TX | Adult | — | 21 | 10– 14 d | CTMax | LOE | 1 °C min^-1^ | 36.28 | 0.57^b^ | 15 | 61 |
|  | San Saba River, TX | Adult | — | 21 | 10– 14 d | CTMax | LOE | 1 °C min^-1^ | 36.35 | 0.32^b^ | 15 | 61 |
|  | Pedernales River, TX | Adult | x̅: 33^a^, max: 39^a^ | 21 | 10– 14 d | CTMax | LOE | 1 °C min^-1^ | 36.02 | 0.36^b^ | 15 | 61 |

Table S1. (Continued)

| Species | Location | Life stage / size | T_ambient_ | T_acclimation_ °C | Acclimation time | Test | Endpoint | ∆ T | Thermal tolerance, °C | | | Reference |
| --- | --- | --- | --- | --- | --- | --- | --- | --- | --- | --- | --- | --- |
|  |  |  |  |  |  |  |  |  | x̅ | SD | n |  |
|  | Cuivre River, Perche Creek, & Lamine River, MO | 0.4–0.2 g | — | 26 | 63–160 d | CTMax | LOE | 2 °C h^-1^ | 38.1 | 0.42 | 11 | 63 |
|  | Platte River, NE | — | x̅ = 23.7 | 23.7 | ≥ 14 d | CTMax | LOE | 1 °C min^-1^ | 37.4 | Range: 35.7–38.7 | 30 | 64 |
|  | **Platte River, NE** | **—** | **x̅ = 23.7** | **23.7** | **≥ 14 d** | **LTMax** | **D** | **1 °C min^-1^** | **39.8** | **Range: 38.1–41.0** | **30** | **64** |
|  | Platte River, NE | — | — (Spring) | Ambient | — | CTMax | LOE | 1 °C min^-1^ | 30.8 | Range: 27.8–33.2 | 40 | 64 |
|  | Platte River, NE | — | x̅ = 24.6 (Summer) | Ambient | — | CTMax | LOE | 1 °C min^-1^ | 37.7 | Range: 35.1–39.0 | 70 | 64 |
|  | Platte River, NE | — | x̅ = 17.9 (Fall) | Ambient | — | CTMax | LOE | 1 °C min^-1^ | 33.7 | Range: 29.0–35.7 | 40 | 64 |
|  | Central Platte River, NE | — | 22.4 | 22.7 ± 7 (fluct.) | ≥ 14 d | CTMax | LOE | 1 °C min^-1^ | 37.6 | 0.5 | 10 | 64 |
|  | Central Platte River, NE | — | 22.4 | 21.7 (stable) | ≥ 14 d | CTMax | LOE | 1 °C min^-1^ | 35.9 | 0.5 | 20 | 64 |
|  | Central Platte River, NE | — | 22.4 | Ambient | — | CTMax | LOE | 1 °C min^-1^ | 37.0 | 0.8 | 20 | 64 |
|  | Central Platte River, NE | — | 25.0 | 25 ± 7 (fluct.) | ≥ 14 d | CTMax | LOE | 1 °C min^-1^ | 38.9 | 0.3 | 10 | 64 |
|  | Central Platte River, NE | — | 25.0 | 25.5 (stable) | ≥ 14 d | CTMax | LOE | 1 °C min^-1^ | 37.5 | 0.7 | 10 | 64 |
|  | Central Platte River, NE | — | 25.0 | Ambient | — | CTMax | LOE | 1 °C min^-1^ | 37.7 | 0.7 | 20 | 64 |
|  | Lower Platte River, NE | — | 25.0 | 25.3 ± 7 (fluct.) | ≥ 14 d | CTMax | LOE | 1 °C min^-1^ | 37.1 | 0.4 | 10 | 64 |
|  | Lower Platte River, NE | — | 25.0 | 25.3 (stable) | ≥ 14 d | CTMax | LOE | 1 °C min^-1^ | 36.9 | 0.6 | 10 | 64 |
|  | Lower Platte River, NE | — | 25.0 | Ambient | — | CTMax | LOE | 1 °C min^-1^ | 36.9 | 0.3 | 10 | 64 |
|  | **Lower Platte River, NE** | **TL: 30–70 mm** | **“warm water”** | **30** | **14 d** | **LTMax** | **D** | **0.3 °C min^-1^** | **40.9** | **0.2^b*^** | **≥12** | **53** |

Table S1. (Continued)

| Species | Location | Life stage / size | T_ambient_ | T_acclimation_ °C | Acclimation time | Test | Endpoint | ∆ T | Thermal tolerance, °C | | | Reference |
| --- | --- | --- | --- | --- | --- | --- | --- | --- | --- | --- | --- | --- |
|  |  |  |  |  |  |  |  |  | x̅ | SD | n |  |
|  | Across the US | — | “warm water” | — | — | MWTT | — | — | 34.0 | — | — | 56 |
| *Cyprinella venusta* | **Blue River, Pennington Creek, & Byrds Mill Spring, OK** | **Adult; TL: 65–90 mm (x̅ = 75)** | **June x̅: 20** | **20** | **≥ 2 w** | **LTMax** | **D** | **2 °C / h** | **36.4** | **—** | **10** | **55** |
|  | **Blue River, Pennington Creek, & Byrds Mill Spring, OK** | **Adult; TL: 54–80 mm** | **June x̅: 20** | **20** | **≥ 2 w** | **CLMax**  **(10–14 d)** | **D** | **1 °C d^-1^** | **39.0** | **—** | **10** | **55** |
| *Moxostoma anisurum* | Across the US | — | “cool water” | — | — | MWTT | — | — | 29.6 | — | — | 56 |
| *Moxostoma erythrurum* | Across the US | — | “cool water” | — | — | MWTT | — | — | 29.6 | — | — | 56 |
|  | **Walholding River, WY** | **Juvenile & small adult; 153–350 mm** | **—** | **20.6–23.8** | **7 d** | **LTMax** | **D** | **4.8 °C h^-1^** | **35.4** | **—** | **10–12 / rep.** | **54** |
| *Moxostoma macrolepidotum* | **Walholding River, WY** | **Juvenile & small adult; 153–350 mm** | **—** | **20.6–23.8** | **7 d** | **LTMax** | **D** | **3.4 °C h^-1^** | **35.1** | **—** | **10–12 / rep.** | **54** |
|  | **Walholding River, WY** | **Juvenile & small adult; 153–350 mm** | **—** | **20.6–23.8** | **7 d** | **CLMax** | **D** | **0.5 °C 12h^-1^** | **33.3** | **—** | **10–12 / rep.** | **54** |
| *Moxostoma robustum* | Oconee River, GA | 30–90 d | — | 20 | ≥ 2 w | CTMax | LOE | 0.3 °C min^-1^ | 34.9 | 3.3^b^ | 12 | 65 |
|  | Oconee River, GA | 30–90 d | — | 30 | ≥ 2 w | CTMax | LOE | 0.3 °C min^-1^ | 37.17 | 0.11 | 11 | 65 |
